# Supplementary material for: Identification and Characterization of a Novel Major Facilitator Superfamily Efflux Pump, SA09310, Mediating Tetracycline Resistance in Staphylococcus aureus
Source: Antimicrob Agents Chemother. 2023 Mar 23;67(4):e01696-22. doi: 10.1128/aac.01696-22 (PMC10112136; doi:10.1128/aac.01696-22)
Supplement: Supplemental file 1 — Supplemental material. Download aac.01696-22-s0001.pdf, PDF file, 0.9 MB [file aac.01696-22-s0001.pdf]

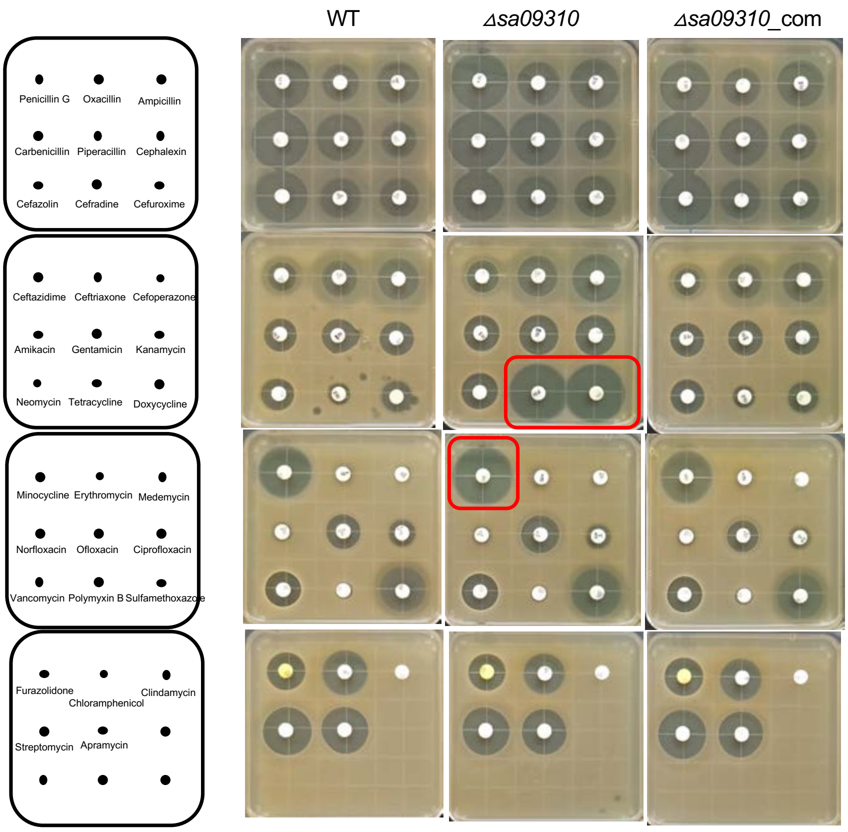

Supplemental Fig. 1. Disk diffusion assay of *S. aureus* WT, *Δsa09310*, and *Δsa09310_com* against different antibiotics. The susceptibility of *S. aureus* USA300 WT, Δ*sa09310*, and Δ*sa09310*_com against 32 different antibiotics was tested by disk diffusion assay. Strain Δ*sa09310* exhibited increased susceptibility to antibiotics tetracycline, doxycycline, and minocycline were highlighted with red squares.

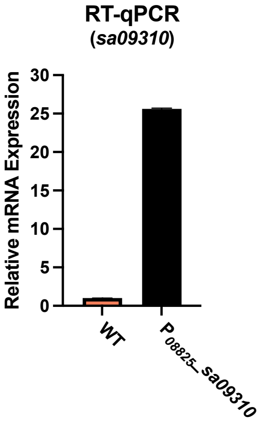

Supplemental Fig. 2. Overexpression of *sa09310*. Overexpression of *sa09310* was achieved by using the replicative vector pQLV1025 with a strong constitutive promoter P_{08825}. RT-qPCR was performed to confirm the overexpression of *sa09310* gene. The expression level of *sa09310* (black bar) in the overexpression strain was relative to WT (orange bar).

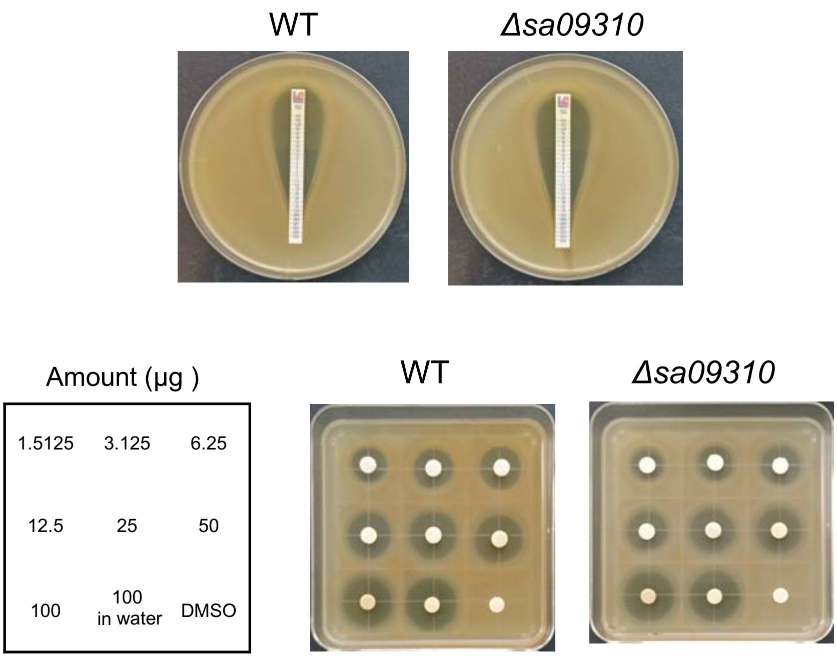

Supplemental Fig. 3. Susceptibility of Δ*sa09310* to tigecycline.

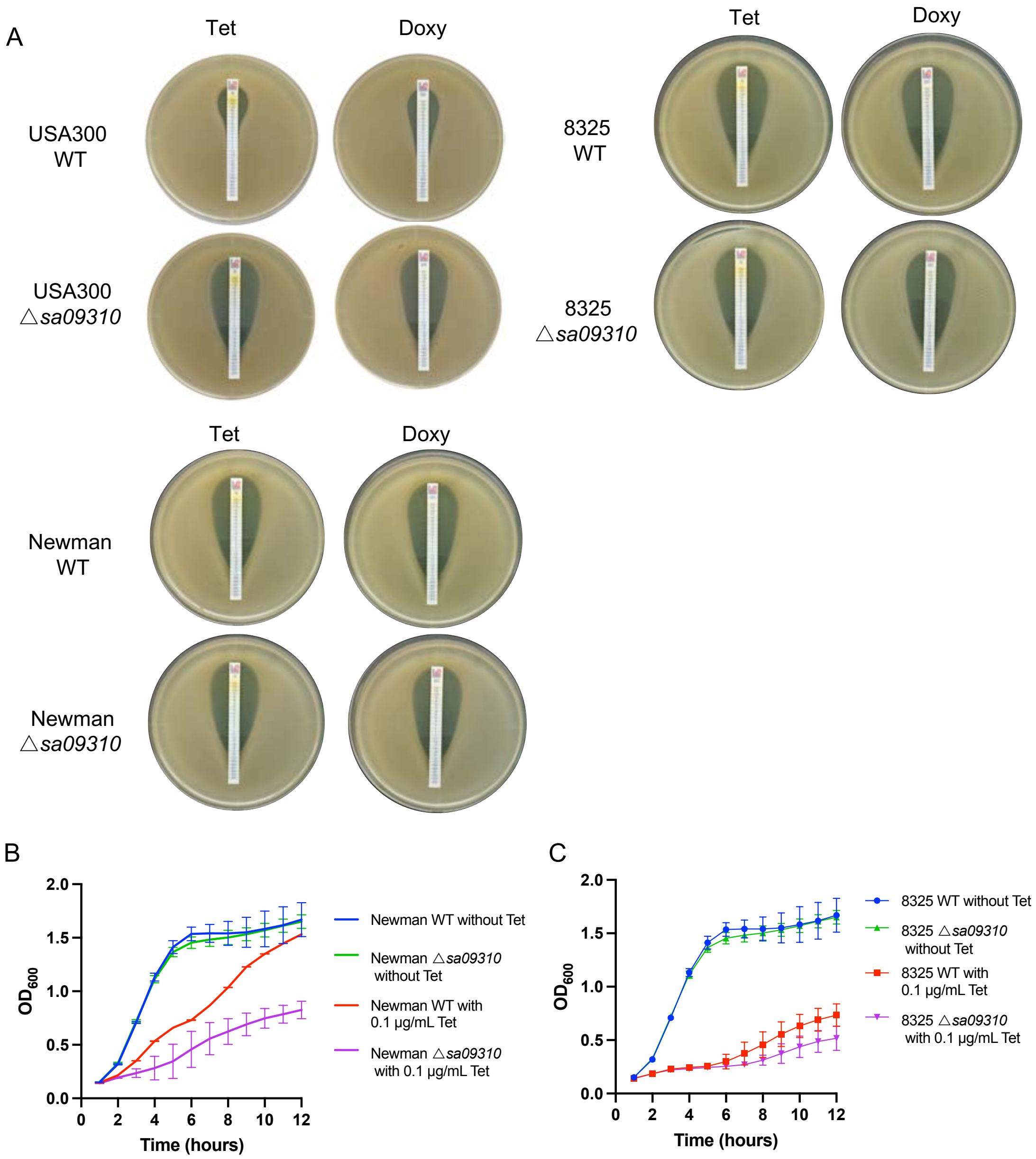

Supplemental Fig. 4. Susceptibility of different *S. aureus* strains to tetracycline and doxycycline. A. E-test to measure the MICs of tetracycline and doxycycline to *S. aureus* USA300, *S. aureus* Newman, *S. aureus* 8325 WT strains and their corresponding SA09310 coding gene null mutants. B. Growth curve of *S. aureus* Newman WT and *sa09310* gene deletion mutant strains in the presence or absence of 0.1 μg/mL tetracycline. C. Growth curve of *S. aureus* 8325 WT and *sa09310* gene deletion mutant strains in the presence or absence of 0.1 μg/mL tetracycline.

Supplemental table 1. Antimicrobial susceptibility of *S. aureus* WT and its mutant strains in disk diffusion assay

Antimicrobial agent	Zone diameter of inhibition (mm[a])		
	WT	Δ*sa09310*	Δ*sa09310*_com
Penicillin G (10 U)	24 ± 0.5	24.5 ± 1	23.5 ± 0.5
Oxacillin (1 μg))	17 ± 0.5	17.5 ± 0.5	17 ± 0.5
Ampicillin (10 μg))	19.5 ± 0.5	20 ± 0.5	21 ± 0.5
Carbenicillin (100 μg)	26 ± 0.5	27 ± 0.5	27.5 ± 0.5
Piperacillin (100 μg)	21.5 ± 0	22.5 ± 0.5	22.5 ± 0.5
Cephalexin (30 μg)	15 ± 0.5	17 ± 0	16.5 ± 0
Cefazolin (30 μg)	24 ± 0.5	23.5 ± 1	24 ± 0.5
Cefradine (30 μg)	19 ± 0	18.5 ± 0.5	18 ± 0.5
Cefuroxime (30 μg)	18 ± 0.5	19 ± 0	18 ± 0
Ceftazidime (30 μg)	13 ± 0.5	14 ± 1	13 ± 0.5
Ceftriaxone (30 μg)	16 ± 0.5	17.5 ± 0.5	16.5 ± 0.5
Cefoperazone (75 μg)	18.5 ± 0	19 ± 0	18 ± 0
Amikacin (30 μg)	14.5 ± 0.5	15.5 ± 0	15.5 ± 0
Gentamicin (10 μg)	15.5 ± 0.5	16 ± 0	16 ± 0
Kanamycin (30 μg)	15.5 ± 0.5	16.5 ± 0.5	16 ± 0
Neomycin (30 μg)	15 ± 0.5	16 ± 1	15.5 ± 0.5
Tetracycline (30 μg)	9.5 ± 0.5	27 ± 0.5	10 ± 0.5
Doxycycline (30 μg)	14 ± 0.5	27 ± 0.5	14 ± 0
Minocycline (30 μg)	24 ± 1	25.5 ± 0.5	24 ± 0.5
Erythromycin (15 μg)	N	N	N
Midecamycin (30 μg)	N	N	N
Norfloxacin (10 μg)	N	N	N
Ofloxacin (5 μg)	15.5 ± 0.5	16.5 ± 1	15 ± 0
Ciprofloxacin (5 μg)	12 ± 0.5	11.5 ± 1	10.5 ± 0.5
Vancomycin (30 μg)	16 ± 0.5	17 ± 0.5	16 ± 0.5
Polymyxin B (300 U)	N	N	N
Sulfamethoxazole (30 μg)	21 ± 0	21 ± 0.5	21 ± 0
Furazolidone (300 μg)	17 ± 0	16.5 ± 0.5	17.5 ± 0
Chloramphenicol (30 μg)	21 ± 1	19 ± 1	19 ± 1
Clindamycin (2 μg)	N	N	N
Streptomycin (1 mg)	22.5 ± 0.5	21.5 ± 1	22 ± 0.5
Apramycin (0.5 mg)	21 ± 0.5	21 ± 0.5	20.5 ± 1

[a] Sensitivities were assessed by measuring the diameters of the zones of growth inhibition in three independent assays, and the means ±1 SD were calculated.

Supplemental table 2. Primers used in this study.

Primer name	Description	Sequence (5' to 3')
QL1165	09310 KO up F	cactcatcgcagtgcagcggGCATGTTCAGGACTTAAGACTAAG
QL1166	09310 KO up R	TGAGTAAATAGAATTAACGTTATAGACTTG
QL1167	09310 KO down F	CAAGTCTATAACGTTAATTCTATTTACTCA<br>ATTTCATAGTTCCTCTCTCTTTTTTGTC
QL1168	09310 KO down R	cttgcatgcctgcaggtcgaCGCAAGCCTTGATAAGAATTTC
QL1169	09310 check F	CATAGGGACGACATTTGCCGC
QL1170	09310 check R	CGGTTAGGACAGTCAAACCC
QL1293	qPCR for *sa09310* F	TGTCGCGGTAACGCAATTTA
QL1294	qPCR for *sa09310* R	TGAAGCGATTGTAGACTCCCA
QL0152	qPCR for 16s rRNA F	GCTCGTGTCGTGAGATGTTGG
QL0153	qPCR for 16s rRNA R	TTTCGCTGCCCTTTGTATTGT
QL1301	*sa09310* overexpression F	CATAAAAAAGGAGACATGCAAATATACCTAAATCAGTCTGGTGG
QL1302	*sa09310* overexpression R	TGGTGATGAGAACCTCTCGAGCATGCATCTATTTTTTTAGGTTGCG

Supplemental table 3. Conservation of SA09310 in different strains of *S. aureus.*

**Strain name**	**Taxonomy ID**	**Homolog identified**	**Accession No.**	**Percent identity to SA09310 (%)**
S. aureus RF122	273036	Yes	WP_001025059.1	99.75
S. aureus subsp. aureus 70	548475	Yes	WP_001025071.1	99.75
S. aureus subsp. aureus COL	93062	Yes	WP_001025061.1	100
S. aureus subsp. aureus ED98	681288	Yes	WP_001025067.1	99.75
S. aureus subsp. aureus JH1	359787	Yes	WP_001025067.1	99.75
S. aureus subsp. aureus JH9	359786	Yes	WP_001025067.1	99.75
S. aureus subsp. aureus MRSA252	282458	Yes	WP_001025064.1	99.75
S. aureus subsp. aureus MSSA476	282459	Yes	WP_001025064.1	99.75
S. aureus subsp. aureus Mu3	418127	Yes	WP_001025067.1	99.75
S. aureus subsp. aureus Mu50	158878	Yes	WP_001025067.1	99.75
S. aureus subsp. aureus Mu50-omega	585891	Yes	WP_001025067.1	99.75
S. aureus subsp. aureus MW2	196620	Yes	WP_001025067.1	99.75
S. aureus subsp. aureus N315	158879	Yes	WP_001025067.1	99.75
S. aureus subsp. aureus NCTC 8325	93061	Yes	WP_001025061.1	100
S. aureus subsp. aureus str. CF-Marseille	505321	No[a]	None	None
S. aureus subsp. aureus str. JKD6008	546342	Yes	WP_001025061.1	100
S. aureus subsp. aureus str. JKD6009	546343	Yes	WP_001025061.1	100
S. aureus subsp. aureus str. Newman	426430	Yes	WP_001025061.1	100
S. aureus subsp. aureus USA300_FPR3757	451515	Yes	WP_001025061.1	100
S. aureus subsp. aureus USA300_TCH1516	451516	Yes	WP_001025061.1	100

[a] There is no proteome information of the strain.
